# Deep Brain Stimulation of the Ventral Intermediate Nucleus of the Thalamus in Writer’s Cramp: A Case Report

**DOI:** 10.5334/tohm.645

**Published:** 2021-11-08

**Authors:** Lisa Hirt, Fabio Grassia, Jeanne Feuerstein, John A. Thompson, Steven Ojemann, Drew S. Kern

**Affiliations:** 1 Department of Neurosurgery, University of Colorado School of Medicine, Aurora, CO, US; 2 Department of Neurology, University of Colorado School of Medicine, Aurora, CO, US; 3 Department of Neurology, Rocky Mountain Regional VA Medical Center, Aurora, CO, US

**Keywords:** writer’s cramp, deep brain stimulation, directional, VIM, thalamus, dystonia

## Abstract

**Background:**

Globus pallidus internus (GPi) deep brain stimulation (DBS) and thalamotomy are interventions for writer’s cramp (WC). Ventralis intermedius nucleus (VIM) DBS is targeted for tremor, however, many aspects of VIM DBS remained underexplored in WC.

**Case Report:**

A 62-year-old man with WC underwent DBS. Dystonic tremor improved intraoperatively with ventralis oralis anterior (VoA)/ventral oralis posterior (VoP) and with subthalamic nucleus stimulation; although greatest benefit was obtained with VIM stimulation. Sustained benefit with VIM DBS at ten months post-operative was obtained.

**Discussion:**

This case demonstrates an intraoperative approach in target selection and supports benefits of VIM DBS for WC.

**Highlights:**

This case highlights the intraoperative approach and clinical effects of VIM DBS in the treatment of medically refractory writer’s cramp (WC). We contextualize our results from this case with previous reports of VoA/VoP stimulation for WC.

## Introduction

Writer’s cramp (WC) is a focal, task specific dystonia characterized by abnormal posturing of the arm and hand that predominately occurs while writing. Current non-surgical treatment options include medications, botulinum toxin injection, and occupational therapy. In a significant subset of patients, these treatments, often lead to inconsistent results with variation in sustained clinical benefit and may result in unwanted adverse effects [1]. In refractory cases, neurosurgical interventions including ablation and deep brain stimulation (DBS) have been performed. Although the pathophysiology of WC remains unknown, dysfunction in the pallido-thalamocortical pathway is strongly implicated [2]. Consequently, surgical targeting of the thalamic nuclei that receive pallidal afferent fibers, specifically the ventralis oralis anterior (VoA) and ventral oralis posterior (VoP), have been widely proposed as the preferred targets [3]. Another thalamic nucleus, the ventralis intermedius nucleus (VIM) that receives cerebellar afferent fibers, is a common target for tremor including dystonic tremor [4,5]. Tremor is a common feature of WC; however, there remain limited reports on the clinical efficacy of VIM targeting for this condition [6]. Finally, prior literature describing thalamic DBS for WC remains sparse and largely composed of case reports as the most common surgical approaches have been ablative [2,3,6,7,8,9,10,11,12,13,14].

In this case report, we present a patient with WC implanted with a directional DBS lead in the VIM. We outline our surgical approach using neurophysiology and intraoperative clinical assessment that aided in targeting and in lead selection. In addition, we provide clinical outcomes with follow-up at 10 months post-operative.

## Case Description

A 62-year-old right-handed man presented to our clinic for advanced treatment options for WC. He had no previous injuries of the limb, no family history of dystonia or tremor. However, he did report repetitive use of the right hand with handwriting and shooting firearms. Initial symptoms occurred in his early 30’s and were characterized by hand cramping and abnormal penmanship when writing. The symptoms were intrusive and unremitting, and over the past nine years significantly interfered with his ability to handwrite with his dominant hand. These symptoms progressed over the past 5 years impairing his ability to perform any action that involved gripping objects. Consequently, he rarely used his right hand for daily activities. He had previously been prescribed tetrabenazine, baclofen, and had undergone botulinum toxin injections without significant benefit or unwanted adverse effects therefore medication was discontinued.

On examination, there was no abnormal posturing or tremor of the left upper limb, lower limbs, neck, face, and voice. At rest there was no abnormal posturing of the right hand. With the right hand held in pronation and full extension, the 2^nd^ and 3^rd^ digit flexed at the metacarpophalangeal joints. This abnormal posturing was also exhibited with the arms held in wing beating position (arms abducted with elbows at level of the shoulders and arms flexed with hand pronated and positioned just under the nose). In addition, in the wing beating position, there was slight flexion of the right wrist. No abnormal posturing was demonstrated in supination. These signs were most obvious with writing, but there was no abnormal posturing evident with the patient holding a writing instrument. However, immediately upon placing the writing instrument onto the paper and initiating writing, a greater extent of flexion of the metacarpophalangeal joints and wrist was evident. With continued writing, the thumb also extended and writing was characterized as having a jerky quality and was illegible. (***Video 1***). No mirrored movements of the contralateral limb were evident.

**Video 1:** **Visualization of patient’s tremor over time.** Demonstration of preoperative, intraoperative, and postoperative handwriting, Archimedes’ spiral, and line drawings.

The consensus treatment plan from the patient care conference was to pursue DBS with surgical targeting of the VoA/VoP complex based upon literature review. Although there is limited data on thalamic stimulation, immediate tremor suppression intraoperatively has been reported. For these reasons, we initially planned on placing a Boston Scientific (Valencia, California) 2201 electrode with 8 vertically and evenly spaced contacts into the thalamus. The intention was that the contacts would span the VoA/VoP. The surgical approach has been previously described [15]. Two microelectrodes (AlphaOmega NeuroProbe) were simultaneously descended to target at 12.46 mm (X), 1.23 mm (Y), and 8.45 mm (Z) (tract 2) and 2 mm anterior from this tract (tract 1).

The first trajectory had minimal microelectrode recording (MER) consistent with thalamic cells. However, intraoperative electrophysiology was most consistent with STN recordings beginning at 2.3 mm above the intended target. Consistent with the motor territory of the STN, kinesthetic responses were evident from 1.6 mm above to –2.3 mm below radiographic derived target. Using the MER electrode that has a collar 3 mm from the tip of the electrode and can be used for stimulation, we tested macrostimulation of the STN at 1.5 mm above the intended target. STN macrostimulation provided an improvement in dystonic posturing and in writing tested by performing Archimedes’ spirals at 0.5 mA. The patient reported paresthesia in the right arm at 1mA that became intolerable and spread into his face as the stimulation intensity increased.

The second trajectory performed simultaneously with the first trajectory, which spanned the targeted brain areas showed kinesthetic responses, specifically active range of motion, during motor testing at 9.4mm, 5.3 mm, 2.3 mm, above intended target. This location was most consistent with the VoP. Macrosimulation at 3 mm and 5.5 mm above target provided less benefit as tested in posturing and in writing as well as greater stimulation adverse effects compared with track one macrostimulation ***Figure 2b***. Given the beneficial effects of STN stimulation, the DBS electrode Boston Scientific 2202 with horizontal directional contacts of the second and third level was implanted with the tip at –3.5 mm below the intended target. Despite the clinical benefits, the patient experienced low threshold for stimulation induced adverse effects at 0.5 mA that was not overcome with various parameter adjustments and use of directional contacts. Consequently, the DBS electrode was removed and a third tract using MER was implanted.

**Figure 1:**
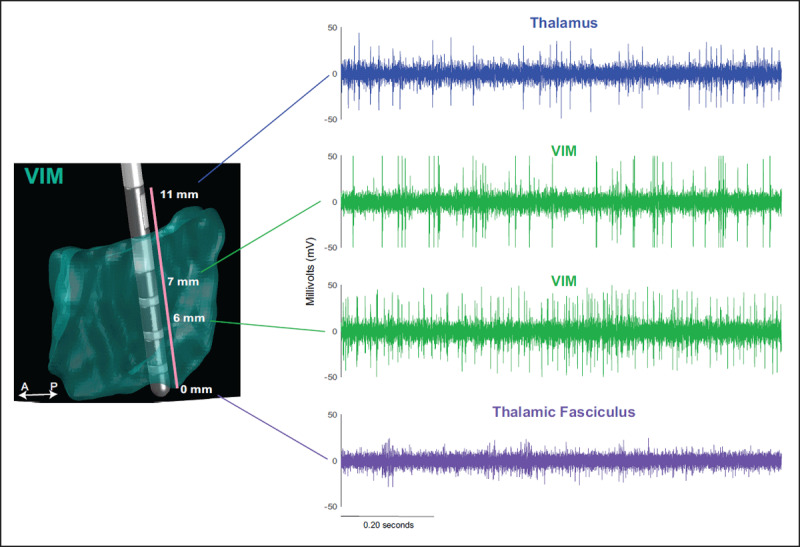
**Intraoperative electrophysiology recordings.** One second interval microelectrode recordings representing the electrophysiology through basal ganglia structures, including the thalamus (blue), VIM (green), and thalamic fasciculus (purple). The lead in the figure provided the authors an objective measure to confirm the depth of the electrophysiology displayed, and demonstrated the scale and location in reference to the VIM where the microelectrode recordings occurred.

**Figure 2:**
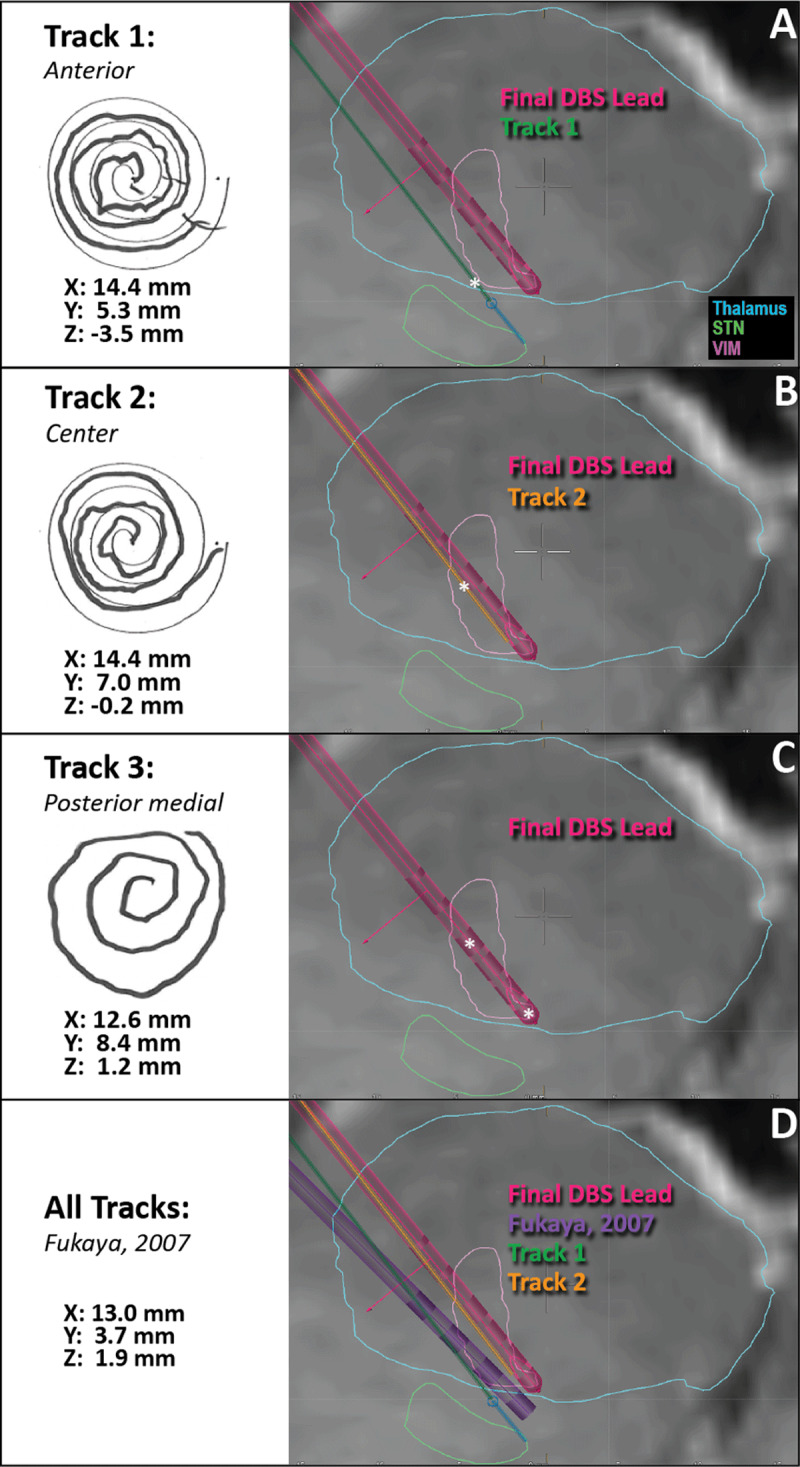
**BrainLab (Munich, Germany) image displaying the three trajectories the patient had implanted during the DBS procedure, and the Vo/VIM DBS trajectory from Fukaya (2007).** The arrow from the terminal lead (pink) observed in panels A – D portrays the directionality of the electrode. Coordinates in panels A–C are in reference to the MCP of our patient, the coordinates in D are the means from the Fukaya trajectory (6). All images are shown at the center of the intended target and all Archimedes’ spirals were conducted intraoperatively. The white asterisks overlaid on each track (panels A–C) represents the location where the Archimedes’ spiral were obtained. **A**: In green is the anterior trajectory from center. The blue track originating from the distal end of the green trajectory represents the implanted electrode into the STN. The tip of the electrode extended –3.5 mm inferior to the MCP into the STN. Panel **B**: The yellow trajectory (center track) was intended to span the targeted brain areas. Comparison of Archimedes’ spirals between panel A and B shows slight clinical improvement in track one. Panel **C**: the pink lead represents the third track that targeted the VIM and produced the best clinical outcomes without adverse effects, as seen by comparison of Archimedes’ spirals. Panel **D**: The Fukaya Vo/VIM electrode (in purple) was overlayed on our three trajectories for comparison of electrode positioning. The electrode was modified based upon their case description by removing the distal contact and made blunt (6).

The third track was implanted at 1.7 mm posterior and 1.7 mm medial of tract 2. Thalamic activity was recorded from 10 mm to 0.8 mm above radiographic target. Kinesthetic responses of passive range of motion of the upper limb were obtained from 4.7 mm to 0.8 mm above radiographic target (***Figure 1***). No clear kinesthetic active range of motion of the upper limb was obtained in this tract consistent with VIM. Given the uncertainty of VoP compared with VIM stimulation, macrostimulation was applied at 7 mm, 4 mm and 2.3 mm above radiographic derived target. Greatest benefit was obtained at 4 mm compared with all macrostimulation tests of all tracts at 1.5 mA (***Figure 2***). Furthermore, no stimulation adverse effects were obtained. Given that less benefit was obtained with more dorsal macrostimulation, the Boston Scientific 2202–45 electrode was implanted with the tip positioned at 0 mm of the intended target. Intraoperative testing of the DBS electrode demonstrated superior clinical benefit with only transient paresthesia up to 2 mA (***Video 1***).

**Figure 3:**
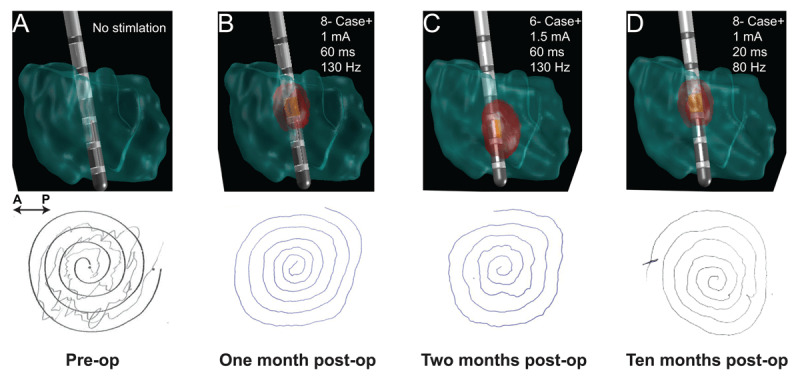
**Comparison Archimedes’ of spirals, stimulation parameters and Volume of Tissue Activation (VTA) in red.** Using preoperative MRI and postoperative CT imaging, the VTA was modeled from patient stimulation parameters and created with LeadDBS [21] and Fastfield [22], which does not take frequency into account. The VTA represents simulation meant to target the VIM, seen as time progresses. Archimedes’ spirals improved as stimulation was applied to the VIM when compared to preoperative measures (panel A). The motor scales remained stable postoperatively at one month (panel B), two moths (panel C), and ten months (panel D). The high overlap of VTA onto the VIM displays that the VIM is primarily stimulated and relates to the improved motor scales demonstrated in panels B–D.

One week post lead implantation, a Boston Scientific IPG battery (Vercise; Valencia, California) was placed in the left pectoral area and cervical extension wires were connected. Programming was initiated one month post lead implantation. and was initially programmed every one to two weeks for the first three programming sessions at University of Colorado Hospital. After the preliminary three programming appointments, his care was transferred to the Veterans Affairs (VA) Hospital where he is seen every four months. To assess efficacy, motor scales were collected in the form of Archimedes spirals, Writer’s Cramp Rating Scale (WCRS), and handwriting samples such as writing his name and the word “sunny” which caused him to continuously write without taking the pen off the paper to elicit dystonic posturing with sustained activity [16,17]. Although not validated, we did perform the WCRS that has been used in previous studies and provides an assessment of severity with higher scores indicative of worse performance [18]. There was a noticeable improvement in the WCRS motor scores from baseline, 18 score, at intraoperative programming, 5 score, and at four weeks post-operative, 4 score. Traditional monopolar review was performed at each level by increasing amplitude in increments of 0.5 mA with other parameters held constant at 60 µs and of 130 Hz. Upon demonstrating greatest benefit with ring mode stimulation of contacts 5–6–7–, monopolar review was then conducted of these individual contacts, which demonstrated monopolar stimulation of 6(–) as well as 8(–) provided marked benefit (***Figure 3***). At 10 months postop, the patient is no longer taking medication for dystonia and his programming settings are: case (+), 8(–), 1 mA, 20 µs, 80 Hz. In a prior programming session, the patient was discharged to test monopolar stimulation of 6– and 8– with separate patient programs. The patient determined that 8– provided slightly greater benefit in tremor control compared with 6–. Testing was performed initially at 60 µs and 130 Hz. Subsequently, a range of frequencies (60 to 225 Hz) were tested. Based upon these findings, 80 Hz provided greatest benefit without inducing adverse effects. Thereafter, further benefit was obtained by increasing the amplitude but at the cost of worsening ataxia and paresthesia. Therefore, pulse width was reduced and the patient was discharged with and gradually increased stimulation to 2mA with his patient programmer.

## Discussion

In this report we describe the surgical approach in selecting VIM as the optimal target, the acute and sustained therapeutic benefit of VIM DBS to treat refractory WC. The use of MER, macrostimulation and clinical assessment was of value in selecting the VIM.

In WC, the primary surgical modality is ablation and there are limited case reports of DBS as a therapeutic treatment. Three of these case reports targeted the GPi and significantly alleviated the symptoms of WC with few instances of recurrence [6,7,19]. Similar clinical benefits were observed with VoA/VoP DBS, and one study reported no significant difference between GPi and VoA/VoP stimulation [7,8]. Only one study has directly compared the GPi and VIM in a single patient with the greatest symptom relief with VIM stimulation [6]. In this study an additional four patients underwent VIM stimulation. In comparison to our trajectory, the predominate location of these contacts were on the border of the VoP/VIM (***Figure 2***). To the best of the authors’ knowledge, this is one of the only reports on VIM DBS for WC.

Interestingly, recent data has indicated the optimal location to stimulate to treat dystonic tremor is on the border of the VoP/VIM [4]. However, when the stimulation between the VoP/VIM versus core VIM stimulation was conducted in our patient, there was a clear difference in clinical benefit by specific targeting of the VIM. Our patient had WC, which may involve different circuitry compared with dystonic tremor indicative of the heterogeneity of dystonia. Lastly, to the best of our knowledge this is the first report of a directional lead used in the treatment of WC. Future investigations can assess the long-term benefits for dystonic tremor patients with VIM DBS, additional benefits provided by directional stimulation, and explore the current and future battery technology in relation to WC [20].
